# Energetic costs increase with faster heating in an aquatic ectotherm

**DOI:** 10.1093/conphys/coad042

**Published:** 2023-06-12

**Authors:** Lucy Harding, Andrew L Jackson, Nicholas Payne

**Affiliations:** Department of Zoology, Trinity College Dublin, D02PN40 Dublin, Ireland; Department of Zoology, Trinity College Dublin, D02PN40 Dublin, Ireland; Department of Zoology, Trinity College Dublin, D02PN40 Dublin, Ireland

**Keywords:** thermal ecology, temperature, respirometry, oxygen consumption, metabolism, climate change

## Abstract

The thermal sensitivity of metabolism is widely studied due to its perceived importance for organismal fitness and resilience to future climate change. Almost all such studies estimate metabolism at a variety of constant temperatures, with very little work exploring how metabolism varies during temperature change. However, temperature in nature is rarely static, so our existing understanding from experiments may not reflect how temperature influences metabolism in natural systems. Using closed-chamber respirometry, we estimated the aerobic metabolic rate of an aquatic ectotherm, the Atlantic ditch shrimp *Palaemonetes varians,* under varying thermal conditions. We continuously measured oxygen consumption of shrimp during heating, cooling and constant temperatures, starting trials at a range of acclimation temperatures and exposing shrimp to a variety of rates of temperature change. In a broad sense, cumulative oxygen consumption estimated from static temperature exposures corresponded to estimates derived from ramping experiments. However, further analyses showed that oxygen consumption increases for both faster heating and faster cooling, with rapid heating driving higher metabolic rates than if shrimp were warmed slowly. These results suggest a systematic influence of heating rate on the thermal sensitivity of metabolism. With influential concepts such as the metabolic theory of ecology founded in data from constant temperature experiments, our results encourage further exploration of how variable temperature impacts organism energetics, and to test the generality of our findings across species. This is especially important given climate forecasts of heat waves that are characterised by both increased temperatures and faster rates of change.

## Teaser

We show that rate of temperature change has a systematic effect on the oxygen consumption of ectotherms and that as temperature increases more rapidly, the rate of oxygen consumption increases. These findings have implications for how we predict ectotherm responses to climate change, for example heat waves.

## Introduction

A broad body of research exists examining the thermal sensitivity of metabolism, and the importance of this relationship reaches into many branches of biology and ecology (Brown *et al.,* 2004). This relationship is particularly paramount for ectotherms as temperature directly affects many facets of their physiology such as metabolism and performance as their body temperatures reflect their environments (Angilletta Jr, 2009). This is even more important given that 99% of species on Earth are ectothermic (Ohlberger, 2013) and that climate change is increasingly altering global temperatures and patterns (Kefford *et al.,* 2022).

An extensive suite of studies have explored the thermal sensitivity of metabolism and revealed many persistent patterns and trends. Firstly, when modelling the increase in metabolic rate with increasing temperature seen in ectotherms, physiologists and ecologists widely used an Arrhenius function (Robinson *et al.,* 1983; Gillooly *et al.,* 2001), an application promoted by Brown *et al.* (2004) in the metabolic theory of ecology (MTE). However, although this could be used to construct the thermal performance curve (Huey and Stevenson, 1979) for many biological rates, it began to receive criticism for its inability to fully account for the varying thermal sensitivity of metabolic rate, and several other models have been proposed to describe this relationship (DeLong *et al.,* 2017; Padfield *et al.,* 2021; Arroyo *et al.,* 2022). A key component of focus in these models is the activation energy (E_a_). Dell *et al.* (2011) showed overwhelming evidence that the mean activation energy for metabolic reactions is 0.65 eV, a value which holds true for the thermal responses of many biological traits. An E_a_ value of ~0.65 eV corresponds to a Q_10_ value in the range of 2–3 (Dell *et al.,* 2011), indicating a doubling or tripling in metabolic rate with every 10°C change in temperature. This understanding of the thermal sensitivity of metabolic rate is widely used to model and predict high-level processes such as predicting ecosystem level responses (Dillon *et al.,* 2010; Rubalcaba *et al.,* 2020), understanding species climate resilience (Vajedsamiei *et al.,* 2021) and predicting population growth under a changing climate (Stearns, 1992; DeLong *et al.,* 2018).

Although the thermal sensitivity of metabolism has been widely studied, research has primarily focused on static temperature experiences (i.e. estimating metabolic rate at a range of constant temperatures) (Enders *et al.,* 2006; Williams *et al.,* 2012; Halsey *et al.,* 2015). However, temperature in nature rarely remains static, and so examining metabolic responses under static thermal conditions may not be an accurate reflection of what is occurring in nature. Thermal variability in the environment can have varying effects on fitness components of organisms, potentially hindering the adaptive capacity (Kingsolver and Buckley, 2020). Therefore, we need to better understand how the thermal sensitivity of metabolism varies when temperature is undergoing change. Understanding this is essential to informing predictive models of ectotherm responses to climate change-induced temperature change (Dillon *et al.,* 2010).

Some previous studies have investigated the effect of dynamic and stable temperature regimes on metabolism of ectotherms (Via, 1985; García-Guerrero *et al.,* 2011; Latournerié *et al.,* 2011; Williams *et al.,* 2012; Lake *et al.,* 2013; Vasseur *et al.,* 2014; Zhang *et al.,* 2015; Killen *et al.,* 2017; Semsar-kazerouni and Verberk, 2018; Guzzo *et al.,* 2019). However, the dynamic temperature scenarios used typically involve stepwise temperature changes (i.e. as opposed to continuous rate of increase or decrease) or oscillating temperature regimes (i.e. multiple increases and decreases in temperature over a period of time). The average metabolic rate of the entire exposure period is then estimated and directly compared to a stable temperature regime. This approach makes it difficult to understand the direct influence of heating or cooling rate on the thermal sensitivity of metabolism. To the best of our knowledge, there exists only one study that measured metabolic rate of a terrestrial insect species *Gryllus pennsylvanicus,* continually under continuous temperature change (Lake *et al.,* 2013), which found mixed results: no significant difference was found in estimates of thermal sensitivity of metabolism between dynamic and stable treatments but, absolute estimates of metabolic rate were higher under dynamic thermal conditions than stable. There could be several reasons to expect rates of temperature change to influence the thermal sensitivity of metabolism, which have been raised previously, for example: (i) cost of acclimation may differ with rate of temperature change (Rohr *et al.,* 2018). Faster rates of change may incur additional energetic costs for ectotherms as they are forced to acclimate to a new temperature environment over a shorter period of time; (ii) the balance between supply and demand of resources may be altered as animals operating at higher temperatures, with higher metabolic rates, demand more resources, but also have a greater capacity to supply resources (Gillooly *et al.,* 2006); and (iii) overall metabolic cost may be impacted by rate of temperature change, specifically increasing metabolic cost with slower rates of temperature change, everything else being equal (Rezende *et al.,* 2011).

Given the inherent complexity of natural systems and the accelerating pace of global warming, there is a pressing need for experimental approaches capable of accurately measuring the thermal response of organisms. In this study, we examined whether the rate of temperature change affects the thermal sensitivity of aerobic metabolism. We do this by conducting closed-chamber respirometry on an aquatic ectotherm, the Atlantic ditch shrimp (*Palaemonetes varians*) (Leach, 1814), to estimate oxygen consumption under varying thermal conditions. Closed-chamber respirometry (which measures gas exchange between an animal and its environment) is widely recognised as a valid proxy for measuring aerobic metabolic rate of aquatic ectotherms (Nelson, 2016; Killen *et al.,* 2017; Guzzo *et al.,* 2019; Vajedsamiei *et al.,* 2021) as the rate of oxygen uptake from the environment is expected to relate stoichiometrically to ATP production rate via mitochondrial oxidative phosphorylation (Clarke and Fraser, 2004; Nelson, 2016; Killen *et al.,* 2021). Previous respirometry experiments on shrimps demonstrated that oxygen consumption generally increases with an increase in temperature: *Litopenaeus stylirostris* (Spanopoulos-Hernández *et al.,* 2005) and *Penaeus chinensis* (Chen and Nan, 1993). Furthermore, *P. varians* has been used in temperature studies in the past (Nielsen and Hagerman, 1998; Palma *et al.,* 2009; Cottin *et al.,* 2010; Oliphant *et al.,* 2011; Ravaux *et al.,* 2012; New *et al.,* 2014), making it a suitable study species. In addition to their value as a model organism, shrimp have considerable ecological value in aquatic food webs, serving both as predator and prey. They serve as a primary food source for many large aquatic organisms, whilst also aiding in scavenging and nutrient cycling, ultimately contributing to the dynamics of aquatic ecosystems.

The findings of this study may have significant implications for understanding how aquatic ectotherms, particularly economically important species such as shrimp, respond to global warming, with findings potentially transferable to other aquatic ectothermic species of similar body sizes and biology to the study species. We aim to contribute insights into how temperature fluctuations affect aerobic metabolism in ectotherms, further strengthening predictions of organismal and population response to environmental stressors in aquatic environments, thereby informing sustainability and conservation strategies.

## Materials & Methods

Closed-chamber respirometry was used to estimate the aerobic metabolic rate of *P. varians* by recording the rate of decline in dissolved oxygen (DO) concentration (mg l^−1^) over time, under varying thermal conditions. Measurements were taken individually on 161 shrimp, with 38 experiencing stable temperature, 67 experiencing heating and 56 experiencing cooling.

*P. varians* is a shallow water shrimp native to Western Europe (Ravaux *et al.,* 2012). *P. varians* is known to inhabit areas where the environmental temperature can seasonally fluctuate from 0 to 33°C (Lofts, 1956; Jefferies, 1964; Healy, 1997; Oliphant *et al.,* 2011)*.* They are known to typically inhabit brackish waters but have been found in very low salinities, including fresh water (Hagerman and Uglow, 1984). These shrimps are often used for fishing bait or as live feed for aquarium fish (Palma *et al.,* 2008a; Palma *et al.,* 2008b; Palma *et al.,* 2009). This species was chosen because it has previously served as a model species for temperature studies (Ravaux *et al.,* 2012) due to their adaptation strategies to temperature (Cottin *et al.,* 2010) and their capacity to tolerate periods of severe hypoxia (Hagerman and Uglow, 1984), tolerating low-oxygen tensions before entering anaerobic respiration (Nielsen and Hagerman, 1998; Peruzza *et al.,* 2018).

## Data Collection

### Acclimation phase

Animals for this experiment were sourced from mainland Europe by a specialist aquarium stockist (Seahorse Aquariums Ltd). They were transported in transparent plastic bags filled with brackish water at ~18°C, with transport from source location to laboratory taking ~6 days. On delivery to the laboratory, animals were removed from the bags and placed in five large buckets. Before experimental treatments, animals were acclimated stepwise to the five desired acclimation temperatures (10, 12, 15, 18 and 21°C) during a 24-h period, using temperature-controlled buckets with a photoperiod of 12 h:12 h light:dark. Shrimp were not fed during this period. Shrimp were then transferred to their corresponding acclimation tanks and fed. The laboratory setup consisted of five aerated tanks (37 × 23.5 × 26 cm) filled with unfiltered, treated tap water (~0.5‰), in a room kept with a 12 h:12 h light:dark phase. Tank water was treated with ‘Organic Aqua Start Up’ water treatment and water quality was maintained with weekly ‘Organic Aqua Fish Care’. Temperature in the tanks was maintained at stable temperatures, controlled by programmable units (Inkbird ITC-310 T-B; constant desired temp ±1.0°C), and shrimp were kept at these stable acclimation temperatures for 2–30 days before experiments commenced. A minimum acclimation phase duration of 2 days was chosen in accordance with previous studies of a similar nature on similar (or the same) species (Berglund and Bengtsson, 1981; Nielsen and Hagerman, 1998; Miller *et al.,* 2002; González-Ortegón *et al.,* 2013). The shrimp were provided with flake fish food approximately every second day. Before respirometry, shrimp were fasted for a minimum of 12 h (average fasting time for stable trials was 26 ± 11 h and for heating/cooling trials was 23 ± 12 h).

### Temperature trials

For the stable measurements, DO concentration was recorded for 1–2 h at five stable treatments corresponding to the acclimation treatments (i.e. 10, 12, 15, 18 and 21°C). For the dynamic temperature treatments, DO concentration was measured under four thermal regimes: (i) heating from 15 to 21°C, (ii) 10 to 21°C, and then cooling from (iii) 15 to 10°C and (iv) 21 to 10°C, with starting and ending temperatures corresponding to acclimation temperatures. These regimes were repeated at four ramping rates (0.0083 °C min^−1^/0.5 °C hr^−1^, 0.0167 °C min^−1^/1 °C hr^−1^, 0.0833 °C min^−1^/5 °C hr^−1^ and 0.1667 °C min^−1^/10 °C hr^−1^), where temperature was increased or decreased continuously. Individual shrimps were removed from the acclimation tanks and placed in 200-ml plastic chambers, filled with water taken from the acclimation tanks. The chambers were then partially submerged in temperature-controlled water baths. The temperature of the water baths was controlled by a programmable unit (Inkbird ITC-310 T-B; desired temp ±0.5°C). Shrimp were allowed to settle in the chambers for 1–2 h before commencing measurements to allow them to recover from handling during transfer. Once settled, a lid was placed on the chamber, a DO probe (Go Direct Optical Dissolved Oxygen Probe) was inserted through an opening in the top of the lid and the chamber was hermetically sealed. Probes were 100% calibrated before the beginning of each experimental phase following the manufacturer calibration procedure. Probes began recording as soon as the chamber was sealed, measuring DO concentration (mg l^−1^), DO saturation (%) and temperature (°C) continuously. Sampling frequency of the probe was varied between temperature treatments due to probe memory limitations; when conducting experiments at λ = 0.0083, 0.0167, 0.0833 and 0.1667°C min^−1^, probes recorded at a frequency of 0.2, 0.5, 1 and 1 sample min^−1^, respectively. All experiments were conducted during the photophase on shrimp that had not been fed for ≥12 h (for more details on acclimation and feeding times, see Supplementary Material, Table S1). Individual shrimps were measured alongside ‘control’ chambers (chambers containing tank water but no shrimp) to record bacterial respiration and later correct the DO concentration data. Chambers were visually shielded from external disturbance during respirometry by the high, opaque walls on the water baths. After data collection, shrimp were euthanised by submersion in 90% ethanol, blotted dry and weighed (recording wet weight ± 0.001 g). Chambers were cleaned with 90% ethanol between all experiments to reduce bacterial growth within.

All work was conducted following the principles of the ‘3Rs’: reduction, refinement and replacement. Animal ethics approval for all works was sought from and approved by Trinity College Dublin School of Natural Sciences (SNS) Research Ethics Committee (project number ‘2021–06 (revised)’).

## Data Analysis

Several animals were removed from the analysis phase due to varying reasons: (i) 105T1021R5 was removed because it was found to have lost the ability to right itself at the end of the experiment, (ii) 28T2110R1 and 13T1021R0.5 died during the experiment, (iii) 37T2110R0.5 and 38T2110R0.5 had too short of a fasting period (5.5 h) and (iv) 122T1521R1 timer was set incorrectly. This resulted in a total of 117 ramping treatments and 38 stable treatments for use in the data analysis phase.

All animals were recorded concurrently with control ‘blank’ chambers, containing water from the same source but no shrimp, to remove the effect of background (bacterial) respiration. A linear regression was fitted to the DO concentration of each blank chamber. The DO concentration for each animal was then corrected by subtracting the negative slope of the blank chamber from the DO concentration raw data, such that.

DO _corrected_ = DO _animal_−(− slope _background respiration_).

As this study used closed-chamber respirometry, over periods of up to several hours, the potential occurrence of hypoxic conditions within the chamber required consideration. González-Ortegón *et al.* (2013) defined hypoxia for *P. varians* (and 5 other shrimp species) to be (DO ≤ 3 mg O_2_ l^−1^) and Berglund and Bengtsson (1981) noted hypoxia occurring ≤3 mg O_2_ l^−1^ for *P. adspersus,* a close relative of *P. varians* (Hagerman and Uglow, 1984). Therefore, a hypoxia threshold of 3 mg O_2_ l^−1^ was used for this experiment. When this threshold was reached in a chamber, the experiment was ended; this occurred in 8 trials (49T1521R0.5, 50T1521R0.5, 52T1521R1, 54T1521R1, 62T1021R0.5, 74T2110R0.5, 78T2110R1 and 97T1021R0.5).

### Modelling

Average metabolic rate (MO_2_) was estimated for each animal held at one of the five stable temperature treatments. MO_2_ was estimated following the methods of Svendsen *et al.* (2016), by fitting a linear model to all individual DO concentration data, extracting the slope ($m$) of this line and then correcting for ‘effective volume’ (volume of the chamber − volume of the animal). To avoid difficulties in measuring the volume of the animal, it is common practice to instead subtract the mass of the animal (in kg) from the volume of the chamber (in l) (Svendsen *et al.,* 2016) because shrimp were assumed to be neutrally buoyant in water, so densities are assumed equivalent and equal to 1. Metabolic estimates were also mass-controlled and allometrically scaled (coefficient = 0.8; Christensen *et al.* (2020)):

$$ {MO}_2=\left({V}_{eff}\right)\left(\frac{1}{mass^{0.8}}\right)(m) $$

where ${MO}_2$ is the metabolic rate in milligrams O_2_ per minute per kilogram, ${V}_{eff}$ is the ‘effective volume’, mass is in kilograms and $m$ is the slope of the line of DO concentration versus time. These MO_2_ values were then plotted against average temperature, and a thermal performance curve (TPC) was fitted using the ‘rTPC’ R package (Padfield and O'Sullivan, 2021; R Core Team, 2022). Several models were chosen for fitting based on their ecological applicability to data of this kind, and the AICc score (used for small sample size, *n* = 37) was calculated (Supplementary Material, Table S1) and used for model selection based on the lowest AICc score. The “Rezende” model (Rezende and Bozinovic, 2019) gave the lowest AICc score and thus was chosen for use. Total performance $pf$ was calculated using the full model:

$$ pf=\left\{\begin{array}{l}\left({Ce}^{\frac{Tln{Q}_{10}}{10}}\right)\ \quad\quad\quad\quad\quad\quad\quad\quad\quad \ if\ T<{T}_{th}\\{}\left({Ce}^{\frac{Tln{Q}_{10}}{10}}\right)\times \left(1-d{\left(T-{T}_{th}\right)}^2\right)\ if\ T>{T}_{th}\end{array}\right. $$

where Q_10_ defines the fold change in performance as a result of increasing the temperature by 10°C, $d$ is a constant controlling the rate of decay from threshold temperature ${T}_{th}$ upwards and Ce is the rate of DO decline in this scenario.

This TPC curve was used to predict the total oxygen consumption (mg O_2_) for animals that experienced heating or cooling with bounds set as the starting (T_1_) and ending temperatures (T_2_) for each individual:

$$ \int_{T2}^{T1} pf=\left\{\begin{array}{l}\left({Ce}^{\frac{Tln{Q}_{10}}{10}}\right)\ \quad \quad \quad \quad \quad \quad \quad \quad \quad \ if\ T<{T}_{th}\\{}\left({Ce}^{\frac{Tln{Q}_{10}}{10}}\right)\times \left(1-d{\left(T-{T}_{th}\right)}^2\right)\ if\ T>{T}_{th}\end{array}\right. $$

The relationship between predicted and observed total O_2_ consumption was investigated to see if the stable temperature trials could be used to predict how much oxygen animals would consume in comparable thermal ranges but under differing rates and directions of temperature change. To do this, the area under the curve was calculated by numerical integration using the ‘integrate’ function in R (Piessens *et al.,* 2011), with upper and lower limits set to the start and end temperature of each individual ramping trial. This integral was then converted to total predicted oxygen consumption (mg) by incorporating duration of the trial and mass of the organism using the following equation:

$$ Total\ predicted\ {O}_2\ consumption=\frac{integral}{\lambda}\left({mass}^{0.8}\right) $$

where λ is the rate of temperature change and mass in kilograms. These predicted values were then plotted against the recorded (or observed) total oxygen consumption. Observed total oxygen consumption was calculated, using the recorded DO concentration data, by subtracting the average of the last five DO concentration values from the average of the first five DO concentration values and dividing by the effective volume. These data were log transformed (to overcome the effect of larger O_2_ measurements being prone to wider uncertainties) and plotted against each other.

To further investigate any potential effect of direction of temperature change, the residuals of the previous plot were calculated (i.e. log of observed O_2_ consumption minus log of predicted O_2_ consumption) and the relationship between these residuals and λ was investigated. A breakpoint regression was fit, using Bayesian inference, using the ‘JAGS’ R package (Plummer *et al.,* 2022).

## Results

**Figure 1:**
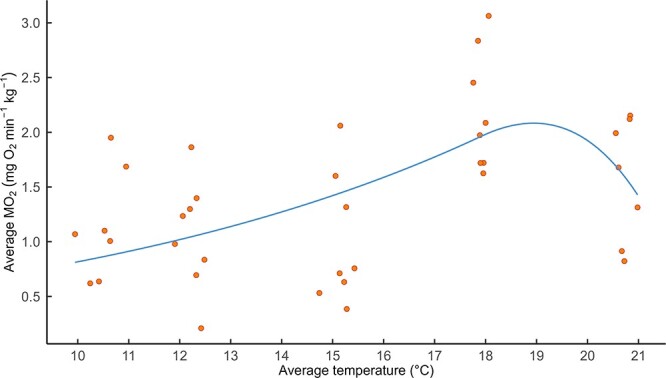
Average MO_2_, for the duration of the trial, against average temperature (°C) for all stable temperature individuals, with thermal performance curve fitted, following the method of Rezende and Bozinovic (2019).

In total, 155 datasets were analysed: 64 heating trials, 53 cooling trials and 38 stable trials, with shrimp wet weight ranging from 0.048 to 0.295 g (Supplementary Material, Table S2). We found that the relationship between MO_2_ and average temperature was best described by a thermal performance curve (Figure 1), specifically that modelled by Rezende and Bozinovic (2019) because it gave the lowest AICc score of 76.83 compared with the second lowest Pawar model AIC of 77.295 (Supplementary Material, Table S2). This Rezende model yielded estimates of 3.03, 0.26, 17.93 and 0.05 for q_10_, Ce, ${T}_{th}$ and $d$, respectively.

**Figure 2:**
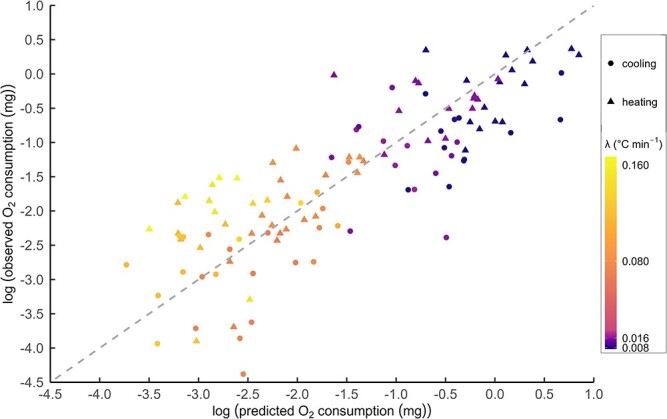
Log observed versus predicted O_2_ consumption (mg) for each ramping individual, with points coloured by rate of temperature change (λ °C min^−1^) and symbols indicating if the temperature was heating (triangle) or cooling (circle). The 1:1 (grey dashed) line indicates where we would expect the points to lie along if the stable treatments perfectly predicted the ramping trial O_2_ consumption.

By integrating under this curve, total predicted oxygen consumption (mg O_2_) was calculated for each individual that underwent heating or cooling (Figure 2). Three individuals (82T2110R10, 84T2110R10 and 36T2110R10) were excluded from this analysis as they showed near zero O_2_ consumption (−0.005, −0.02 and −0.008 mg, respectively), likely due to the fast cooling rate (λ = 0.16°C min^−1^) and short duration of exposure (~60 min).

**Figure 3:**
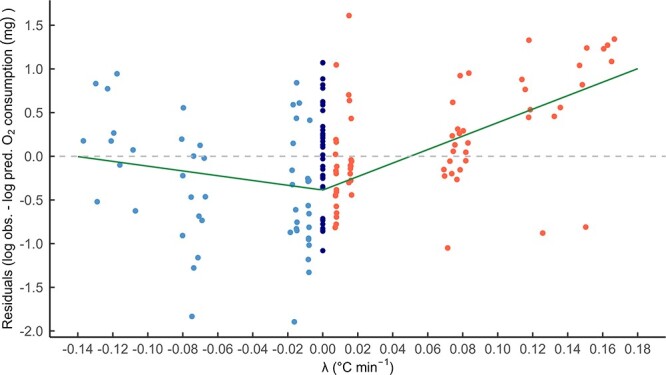
Residuals (i.e. log of observed O_2_ consumption minus log of predicted O_2_ consumption) against rate of temperature change (λ °C min^−1^). Breakpoint regression lines fitted (green solid line). Red points indicate heating trials and blue points indicate cooling trials. The residuals of the stable trials were calculated and overlaid (navy points). A 1:1 identity line is included (grey dashed line).

We see that generally, the points are clustering along the 1:1 line (Figure 2 grey dashed line), indicating that the stable temperature trials predict total oxygen consumption for the dynamic temperature trials. However, any potential effect of direction of temperature change is unclear. Therefore, the residuals of this were calculated (log of observed O_2_ consumption minus log of predicted O_2_ consumption), and the relationship between the residuals and λ was investigated (Figure 3). The breakpoint regression was fit with one breakpoint forced through λ = 0 because all points greater than zero related to heating trails and all points less than zero related to cooling trails.

Figure 3 reveals that faster rates of heating and cooling tend to increase total oxygen consumption. However, there appears to be a weaker influence of rate of cooling than rate of warming on total oxygen consumption: the slope of the heating data and cooling data are significantly greater than zero (slope_heating_ = 7.71, P(slope > 0) = 1.00 for heating, and slope_cooling_ = −2.73, P(slope < 0) = 0.96 for cooling data) with cooling rate having a weaker influence on total oxygen consumption than heating rate.

Furthermore, as temperature increases more rapidly, there is an increasing rate of oxygen consumption (as the regression line crosses the grey dashed line, with a continual positive slope). In addition, animals consumed less oxygen when experiencing slow rates of temperature change than when they were experiencing no change in temperature (evident in the heating and cooling data points, from λ = −0.02 to 0.02). By examining this relationship (Figure 3) between the residuals and λ, it is evident that at slower rates, the stable temperature trials will tend to underestimate oxygen consumption of the dynamic temperature trials (evidenced by the larger majority of data points lying below the grey dashed line), and at faster rates, the predictions will be overestimated (evidenced by the larger majority of data points lying above the grey dashed line).

## Discussion

We show that the rate of temperature change has a systematic effect on the oxygen consumption of a model aquatic ectothermic species. Perhaps most compellingly, we find that as temperature increases more rapidly, the rate of oxygen consumption increases, and although we find a similar relationship as temperature decreases more rapidly, the rate of cooling had less of an influence on the rate of oxygen consumption than the rate of warming. We also see that at slower rates of temperature change, the animals consumed less oxygen than when they were experiencing no change in temperature (i.e. stable temperature). These findings are somewhat unexpected as previous literature indicates that dynamic or variable temperature environments are more energetically costly to ectotherms than stable environments (Williams *et al.,* 2012; Morón Lugo *et al.,* 2020), following Jensen's inequality (Ruel and Ayres, 1999), and that metabolic costs increase with decreasing heating rate, likely due to extended exposure duration (Chown *et al.,* 2009; Rezende *et al.,* 2011). However, these differing findings may be related to Q_10_ values. Chown *et al.* (2009) assume a constant Q_10_ value throughout the temperature change, but Rezende *et al.* (2011) and Gillooly *et al.* (2001) highlight that it is more thermodynamically correct when Q_10_ is expressed as a function of (and changes with) temperature.

Several tentative explanations arise to explain why faster rates of temperature change imply higher metabolic costs. Previous studies have shown that accumulated metabolic costs of individuals under different ramping regimes are not equal, and animals undergoing slower rates of temperature change may be undergoing acclimation due to the associated longer exposure durations (Rezende *et al.,* 2011). Acclimation capacity may be allowing the animals to cope with the increased temperature (Vasseur *et al.,* 2014; Rohr *et al.,* 2018) and to downregulate the increase in metabolism. Furthermore, the longer experiments (i.e. those with the slowest rates of temperature change) may be providing enough time for hardening to occur, a form of phenotypic plasticity (Hoffmann *et al.,* 2003) that protects cells from subsequent injury (Overgaard *et al.,* 2006). Another potential explanation could be that there may be a mismatch between metabolic supply and demand as the temperature increases and the rate of temperature change increases. This could result in internal entropy, causing stress and/or damage, resulting in negative implications for performance over time (Pörtner, 2012; Ritchie, 2018; Vajedsamiei *et al.,* 2021). Future studies to identify the mechanisms underlying the observed increase in metabolic costs at faster rates of temperature change could include: (i) investigating the genetic expression of heat shock proteins and their role in the hardening processes; (ii) energy budget modelling of organisms undergoing temperature change to assess potential mismatches in metabolic supply and demand; and (iii) exploring the potential generality of this identified relationship in other aquatic ectotherms with differing acclimation abilities and of varying body sizes, with the latter allowing for exploration of differing responses to thermal stress across body sizes.

As discussed in the Introduction, there exists only one study that measures metabolic rate continually under continuous temperature change. Lake *et al.* (2013) carried out closed-chamber respirometry on a species of terrestrial insect experiencing dynamic temperature change. Our study was conducted in a similar manner to that of Lake *et al.* (2013). They found that, although estimates of thermal sensitivity of metabolism did not differ significantly between dynamic and stable treatments, absolute estimates of metabolic rate were higher under dynamic thermal conditions than stable ones. Specifically, they reported significantly higher estimated oxygen consumption during cooling at a rate of 0.1°C min^−1^ compared with stable temperature treatments. This finding, however, only occurred in the group of animals that had undergone repeated respirometry and did not occur in animals that were only tested once (as is the case with our study). This discrepancy led them to suggest that rate and direction of temperature change effect on metabolic rate is an aspect that needs to be considered when extrapolating from laboratory studies to the field. At least in a broad sense, our findings concur with those of Lake *et al.* (2013) and confirm that a rapid increase or decrease in ambient temperature results in a greater metabolic demand compared with situations of gradual and slow change. Furthermore, it is noteworthy that Lake *et al.* (2013) estimated oxygen consumption from the air, in contrast to this study, which examined an aquatic (marine) model organism.

A major consideration we acknowledge is the potential for a lag between the water temperature and the body temperature of the animals, and so to counteract this, we intentionally selected a small-bodied aquatic animal (with body wet weight ranging from 0.048 to 0.295 g), therefore increasing the potential for a large heat transfer coefficient and reducing the lag between body and water temperature. Notwithstanding this, should there have been a small lag present during our experiment, this would only increase the effect we show because it would mean that the higher metabolic rates are occurring at even slower rates of heating and cooling because the body would heat and cool slower than the water, thereby increasing the slope of the heating and cooling relationships seen in Figure 3, implying that the impact of λ could be even more influential.

If the dependence of metabolism on heating rate that we document proves to be a general phenomenon seen across species, this would have implications for how we predict ectotherm responses to climate change. The magnitude, duration and frequency of extreme thermal events (i.e. heat waves) is predicted to increase (Coumou and Rahmstorf, 2012; Kefford *et al.,* 2022), and ectotherms are likely more vulnerable to changing temperatures as their body temperature follows that of their environment (Angilletta Jr, 2009; Åsheim *et al.,* 2020). With heat waves increasing in frequency and intensity (Perkins *et al.,* 2012; Minuti *et al.,* 2021), our findings indicate that perhaps heat waves with faster rates of heating may be more costly to the animals than slower heat waves, even if they ultimately reach the same maximum temperature. This potential impact may be further exacerbated given the widespread deoxygenation of both oceanic and inland waters resulting from global warming (Oschlies *et al.,* 2018; Jane *et al.,* 2021). Furthermore, these findings highlight the potential impact of thermal pollution on aquatic ectotherms where discharges of heated effluents, such as sewage, or stagnant water from dams may pose a significant threat to aquatic ectotherms through acute temperature changes. This study is an important step in providing data on ectotherm capacity to adapt to change, through experimental manipulations, and feeding this into mechanistic models for predicting ectotherm responses to climate change and thermal pollution. Our results suggest a systematic influence of temperature change on energetics and encourage future work to determine the generality of this finding across species.

## Acknowledgments

The authors like to acknowledge Dr Jean-Francois Arnoldi, Dr Pepijn Luijckx, Grace McNicholas and Sam Preston for all the their advice, time and effort along the way.

## Funding

This work was supported by Science Foundation Ireland [grant number 18/SIRG/5549 to N. P.] and the Irish Research Council [grant number IRCLA/2017/186 to A. L. J.].

## Conflicts of Interest

The authors know of no conflicts of interest associated with this publication.

## Data Availability

The data underlying this article are available in its online supplementary material and are deposited in the Zenodo Digital Repository (DOI: 10.5281/zenodo.7971310).

## Author Contributions

L.H. and N.P. conceived the ideas and designed methodology; L.H. collected the data; L.H., A.L.J and N.P. analysed the data and L.H. led the writing of the manuscript. All authors contributed critically to the drafts and gave final approval for publication.

## Supplementary Material

Web_Material_coad042
