# Supplementary material for: Energetic costs increase with faster heating in an aquatic ectotherm
Source: Conserv Physiol. 2023 Jun 12;11(1):coad042. doi: 10.1093/conphys/coad042 (PMC10660381; doi:10.1093/conphys/coad042)
Supplement: Web_Material_coad042 [file web_material_coad042.pdf]

Supplementary Information: Energetic costs increase with faster heating in an aquatic ectotherm.

Table S1: Thermal performance curve models fitted to stable temperature data using rTPC r package (Padfield and O'Sullivan, 2021) and associated AICc scores.

**Thermal performance curve model**	**AICc score**
Beta (Niehaus et al., 2012)	81.664
Boatman (Boatman et al., 2017)	81.999
DeLong enzyme-assisted Arrhenius (DeLong et al., 2017)	84.642
Flinn (Flinn, 1991)	77.903
Gaussian (Lynch and Gabriel, 1987)	79.088
Pawar (Kontopoulos et al., 2018)	77.295
Rezende (Rezende and Bozinovic, 2019)	76.838
Sharpe-Schoolfield model (high temperature inactivation only) (Schoolfield et al., 1981)	77.296
Sharpe-Schoolfield model (low temperature inactivation only) (Schoolfield et al., 1981)	82.060
Spain (Spain, 1982)	77.542

Table S2: Experimental data for all temperature trials and individuals, where in Direction, H is heating, C is cooling and S is stable.

**ID**	**Wet weight (g)**	**Fasting period (hours)**	**Acclimation duration (days)**	**Thermal range (°C)**	**Direction**	**λ (°C/min)**	**Mean temperature (°C)**
10T1521R10	0.071	24	23	6	H	0.117826	NA
110T1021R10	0.100	75	25	11	H	0.162769	NA

111T1510R1	0.224	21	26	5	C	-0.01755	NA
114T1510R5	0.178	28.5	26	5	C	-0.07135	NA
117T1521R0.5	0.152	15.5	27	6	H	0.008	NA
118T1521R0.5	0.140	15.5	28	6	H	0.007896	NA
119T1510R0.5	0.214	15.5	28	5	C	-0.00833	NA
11T1521R10	0.138	24.5	9	6	H	0.118605	NA
121T1510R0.5	0.151	15.5	28	5	C	-0.00837	NA
12T1521R10	0.100	24.5	9	6	H	0.117674	NA
15T1021R0.5	0.076	15	4	11	H	0.007515	NA
18T1021R1	0.138	13	11	11	H	0.015994	NA
19T1021R5	0.235	18	21	11	H	0.074571	NA
1T1521R0.5	0.122	13	6	6	H	0.007802	NA
20T1021R5	0.092	18	21	11	H	0.077122	NA
24T1021R10	0.088	19	5	11	H	0.148358	NA
25T1510R0.5	0.176	15	6	5	C	-0.0079	NA
27T2110R0.5	0.170	26	7	11	C	-0.00791	NA
2T1521R0.5	0.133	13	6	6	H	0.007668	NA
30T2110R1	0.108	21	19	11	C	-0.01547	NA
32T2110R5	0.133	19	17	11	C	-0.07524	NA
34T2110R10	0.295	57	16	11	C	-0.1071	NA
39T1510R0.5	0.195	15	6	5	C	-0.0081	NA
3T1521R0.5	0.075	22	4	6	H	0.007067	NA
41T1510R1	0.241	12	4	5	C	-0.01856	NA
42T1510R1	0.090	21	5	5	C	-0.01507	NA
43T1510R5	0.105	14.5	10	5	C	-0.07077	NA
46T1510R10	0.179	17	5	5	C	-0.116	NA

47T1510R10	0.132	20.5	5	5	C	-0.12895	NA
48T1510R10	0.175	27	5	5	C	-0.11947	NA
49T1521R0.5	0.125	30	9	6	H	0.007532	NA
4T1521R1	0.167	20	7	6	H	0.016457	NA
50T1521R0.5	0.077	30	9	6	H	0.007704	NA
51T1521R0.5	0.088	24	10	6	H	0.007302	NA
52T1521R1	0.128	20	7	6	H	0.014839	NA
54T1521R1	0.071	15	12	6	H	0.015	NA
55T1521R5	0.097	27	3	6	H	0.075526	NA
56T1521R5	0.126	27	3	6	H	0.06962	NA
57T1521R5	0.070	27	3	6	H	0.072658	NA
58T1521R10	0.077	24.5	9	6	H	0.11381	NA
59T1521R10	0.080	24.5	9	6	H	0.115714	NA
60T1521R10	0.070	16	12	6	H	0.160571	NA
61T1021R0.5	0.117	15	4	11	H	0.007676	NA
62T1021R0.5	0.115	27	5	11	H	0.007281	NA
63T1021R0.5	0.197	27	5	11	H	0.007785	NA
64T1021R1	0.141	13	11	11	H	0.016024	NA
65T1021R1	0.189	13.5	11	11	H	0.015798	NA
66T1021R1	0.138	13.5	11	11	H	0.015798	NA
67T1021R5	0.107	14.5	7	11	H	0.078507	NA
68T1021R5	0.095	14.5	7	11	H	0.078507	NA
69T1021R5	0.150	16	10	11	H	0.074015	NA
6T1521R1	0.077	20	7	6	H	0.016457	NA
71T1021R10	0.085	20	9	11	H	0.150882	NA
72T1021R10	0.136	20	9	11	H	0.150294	NA
73T2110R0.5	0.171	26	7	11	C	-0.00788	NA

74T2110R0.5	0.093	26	7	11	C	-0.00744	NA
75T2110R0.5	0.090	26	7	11	C	-0.00785	NA
80T2110R5	0.215	22	10	11	C	-0.08061	NA
81T2110R5	0.138	22	10	11	C	-0.08015	NA
82T2110R10	0.135	16	7	11	C	-0.14194	NA
83T2110R10	0.236	22.5	9	11	C	-0.1416	NA
84T2110R10	0.103	17.5	12	11	C	-0.12861	NA
85T1510R0.5	0.094	15	6	5	C	-0.00787	NA
88T1510R1	0.112	21	5	5	C	-0.01507	NA
91T1510R5	0.115	14.5	10	5	C	-0.07385	NA
92T1510R5	0.184	20	10	5	C	-0.07478	NA
93T1510R5	0.150	20	10	5	C	-0.07014	NA
94T1510R10	0.117	15	12	5	C	-0.12095	NA
95T1510R10	0.077	15	12	5	C	-0.11762	NA
96T1510R10	0.151	19	12	5	C	-0.12976	NA
97T1021R0.5	0.104	25.5	9	11	H	0.007709	NA
98T1021R5	0.114	16	10	11	H	0.073723	NA
99T1521R0.5	0.175	24	10	6	H	0.007275	NA
108T1021R1	0.105	16	26	11	H	0.016188	NA
100T1521R10	0.118	16	12	6	H	0.166667	NA
103T1021R5	0.087	71	25	11	H	0.083492	NA
104T1021R5	0.257	71	25	11	H	0.082992	NA
112T1521R5	0.143	26.5	26	6	H	0.081765	NA
113T1521R5	0.122	26.5	26	6	H	0.080286	NA
116T1521R0.5	0.108	15.5	27	6	H	0.00781	NA
120T1521R5	0.231	23	27	6	H	0.076667	NA
123T1521R10	0.100	15	29	6	H	0.125714	NA

14T1021R0.5	0.219	27	22	11	H	0.007742	NA
16T1021R1	0.075	15.5	21	11	H	0.01491	NA
17T1021R1	0.145	15.5	21	11	H	0.015663	NA
21T1021R5	0.208	21	4	11	H	0.078088	NA
22T1021R10	0.156	42	20	11	H	0.132329	NA
23T1021R10	0.134	16	21	11	H	0.135775	NA
53T1521R1	0.129	20	7	6	H	0.01522	NA
5T1521R1	0.091	17.5	23	6	H	0.016243	NA
70T1021R10	0.082	14.5	7	11	H	0.146765	NA
7T1521R5	0.138	15.5	23	6	H	0.074286	NA
8T1521R5	0.133	15.5	23	6	H	0.069231	NA
9T1521R5	0.081	27	3	6	H	0.071389	NA
106T1021R5	0.248	73	25	11	H	0.081732	NA
107T1021R10	0.131	75	25	11	H	0.165079	NA
102T1510R10	0.160	19	12	5	C	-0.123	NA
101T2110R10	0.106	17.5	12	11	C	-0.13684	NA
31T2110R5	0.091	16	17	11	C	-0.06902	NA
29T2110R1	0.104	17.5	18	11	C	-0.01627	NA
115T1510R5	0.124	28.5	26	5	C	-0.07969	NA
45T1510R5	0.234	15.5	23	5	C	-0.06778	NA
33T2110R5	0.048	21	19	11	C	-0.07375	NA
89T1510R1	0.178	15	9	5	C	-0.01694	NA
44T1510R5	0.192	15.5	23	5	C	-0.06712	NA
90T1510R1	0.125	15	9	5	C	-0.01694	NA
40T1510R1	0.125	17.5	23	5	C	-0.01342	NA

79T2110R5	0.153	17	7	11	C	-0.08	NA
35T2110R10	0.232	57	16	11	C	-0.10844	NA
86T1510R0.5	0.162	24	8	5	C	-0.00826	NA
87T1510R0.5	0.172	24	8	5	C	-0.00856	NA
26T1510R0.5	0.195	15	6	5	C	-0.0081	NA
36T2110R10	0.117	22.5	9	11	C	-0.14528	NA
77T2110R1	0.085	25	5	11	C	-0.01481	NA
76T2110R1	0.103	25	5	11	C	-0.01475	NA
78T2110R1	0.158	24	8	11	C	-0.01495	NA
109T1510R1	0.193	21	26	5	C	-0.01738	NA
s27_stable14_15	0.096	14	22	NA	S	NA	15.1
s28_stable14_15	0.190	14	22	NA	S	NA	14.7
s29_stable15_15	0.050	23.5	22	NA	S	NA	15.2
s30_stable15_15	0.199	23.5	22	NA	S	NA	15.1
s31_stable16_18	0.090	18	26	NA	S	NA	18.0
s32_stable17_18	0.154	20	26	NA	S	NA	18.0
s33_stable18_12	0.258	40	27	NA	S	NA	12.2
s34_stable19_12	0.163	42	27	NA	S	NA	12.2
s35_stable20_12	0.127	44	27	NA	S	NA	12.3
s36_stable21_12	0.086	14	28	NA	S	NA	11.9
s37_stable22_12	0.160	21	30	NA	S	NA	12.1
s38_stable23_12	0.119	25	2	NA	S	NA	12.5
s39_stable23_12	0.115	25	2	NA	S	NA	12.3
s40_stable24_12	0.100	25	2	NA	S	NA	12.4

s41_stable25_18	0.118	28	2	NA	S	NA	17.9
s42_stable25_18	0.110	28	2	NA	S	NA	17.8
s43_stable26_18	0.123	28	2	NA	S	NA	17.9
s44_stable26_18	0.139	28	2	NA	S	NA	18.1
s45_stable27_18	0.158	30	2	NA	S	NA	18.0
s46_stable27_18	0.117	30	2	NA	S	NA	17.9
s47_stable28_21	0.116	21	3	NA	S	NA	21.0
s48_stable28_21	0.229	21	3	NA	S	NA	20.8
s51_stable30_10	0.133	23	3	NA	S	NA	10.2
s52_stable30_10	0.113	23	3	NA	S	NA	10.7
s53_stable31_21	0.216	23	3	NA	S	NA	20.7
s54_stable31_21	0.125	23	3	NA	S	NA	20.6
s55_stable32_21	0.215	26	3	NA	S	NA	20.7
s56_stable32_21	0.143	26	3	NA	S	NA	20.6
s57_stable33_10	0.142	26.5	3	NA	S	NA	10.4
s59_stable34_10	0.115	29	3	NA	S	NA	10.6
s60_stable34_10	0.131	29	3	NA	S	NA	10.9
s61_stable35_10	0.129	29	3	NA	S	NA	9.9
s62_stable35_10	0.087	29	3	NA	S	NA	10.5
s63_stable36_15	0.165	13	3	NA	S	NA	15.2
s64_stable36_15	0.163	13	3	NA	S	NA	15.3
s65_stable37_15	0.173	13	3	NA	S	NA	15.4
s66_stable37_15	0.138	13	3	NA	S	NA	15.3
s67_stable38_21	0.160	71	25	NA	S	NA	20.8

Citations

Boatman, T. G., Lawson, T. & Geider, R. J. (2017) A Key Marine Diazotroph in a Changing Ocean: The Interacting Effects of Temperature, CO2 and Light on the Growth of *Trichodesmium erythraeum* IMS101. *PLoS One,* 12**,** e0168796. doi: 10.1371/journal.pone.0168796

Delong, J. P., Gibert, J. P., Luhring, T. M., Bachman, G., Reed, B., Neyer, A. & Montooth, K. L. (2017) The combined effects of reactant kinetics and enzyme stability explain the temperature dependence of metabolic rates. *Ecol. Evol.,* 7: 3940-3950.

Flinn, P. W. (1991) Temperature-Dependent Functional Response of the Parasitoid *Cephalonomia waterstoni* (Gahan) (Hymenoptera: *Bethylidae*) Attacking Rusty Grain Beetle Larvae (Coleoptera: *Cucujidae*). *Environ. Entomol.,* 20: 872-876.

Kontopoulos, D. G., García-Carreras, B., Sal, S., Smith, T. P. & Pawar, S. (2018) Use and misuse of temperature normalisation in meta-analyses of thermal responses of biological traits. *PeerJ,* 6**,** e4363. doi : 10.7717/peerj.4363

Lynch, M. & Gabriel, W. (1987) Environmental Tolerance. *Am. Nat.,* 129: 283-303.

Niehaus, A. C., Angilletta, M. J., Jr, Sears, M. W., Franklin, C. E. & Wilson, R. S. (2012) Predicting the physiological performance of ectotherms in fluctuating thermal environments. *J. Exp. Biol.,* 215: 694-701.

Padfield, D. & O'sullivan, H. (2021) rTPC: Functions for Fitting Thermal Performance Curves: R package version 1.0.2.

Rezende, E. L. & Bozinovic, F. (2019) Thermal performance across levels of biological organization. *Philos. Trans. R. Soc. B: Biol. Sci.,* 374**,** 20180549. doi:10.1098/rstb.2018.0549

Schoolfield, R. M., Sharpe, P. J. & Magnuson, C. E. (1981) Non-linear regression of biological temperature-dependent rate models based on absolute reaction-rate theory. *J Theor Biol,* 88: 719-31.

Spain, J. (1982) BASIC microcomputer models in biology. Adison. Wesley Publishing Co., Reading, Mass.
